# [Retracted] Songorine suppresses cell growth and metastasis in epithelial ovarian cancer via the Bcl-2/Bax and GSK3β/β-catenin signaling pathways

**DOI:** 10.3892/or.2026.9085

**Published:** 2026-02-25

**Authors:** Hongxia Zhang, Ruifeng Dong, Ping Zhang, Yankui Wang

Oncol Rep 41: 3069–3079, 2019; DOI: 10.3892/or.2019.7070

Following the publication of the above paper, it was drawn to the Editor's attention by a concerned reader that, in addition to the duplication of data within Fig. 3A (the 0 h wound-healing assay experimental panels), certain of the immunohistochemical data featured in Fig. 6B were strikingly similar to data that have already appeared in an article in the journal *Oncotarget* that was written by different authors at different research institutes. A subsequent analysis of the data in this paper revealed that certain of the cell invasion assay data in Fig. 3B, western blot data in Figs. 4 and 6, and the histological data showing H&E staining in Fig. 6B had also appeared in articles published in other journals that were written by different authors at different research institutes, one of which has since been retracted.

Owing to the fact that certain of the abovementioned data had already been published prior to the receipt of this article at *Oncology Reports*, the Editor has decided that this paper should be retracted from the Journal. The authors were asked for an explanation to account for these concerns, but the Editorial Office did not receive a reply. The Editor apologizes to the readership for any inconvenience caused.

